# Knowledge and Awareness of Diabetes and Diabetic Retinopathy among Patients Seeking Eye Care Services in Madang Province, Papua New Guinea

**DOI:** 10.1155/2022/7674928

**Published:** 2022-06-01

**Authors:** Bismark Owusu-Afriyie, Anne Caleb, Lorraine Kube, Theresa Gende

**Affiliations:** ^1^Eye Care Programme, Faculty of Medicine and Health Sciences, Divine Word University, Madang, Papua New Guinea; ^2^Fred Hollows Foundation NZ, Auckland, New Zealand

## Abstract

**Purpose:**

To assess the knowledge and awareness of diabetes and diabetic retinopathy among ophthalmic patients in Madang.

**Materials and Methods:**

This was a hospital-based study conducted at Madang Provincial Hospital Eye Clinic in Papua New Guinea. The study included all patients who visited the facility during the period of the study. A structured questionnaire was used to collect data on the patients' demographics and their knowledge and awareness about diabetes and diabetic retinopathy.

**Results:**

A total of 203 (97.6%) patients consented and participated in the study out of 208 patients who were approached. The age of participants ranged from 19 to 78 years with a median (IQR) of 41 (53–29) years. 107 (52.7%) were female participants. A majority of the participants (62.6%) had at least secondary education. A few of the participants (3.9%) had known diabetes, and 134 (66%) had no relatives or friends with diabetes. A total of 145 (71.4%) participants knew that diabetes can affect the eye. Most of the participants (93.6%) checked their eyes only when their vision was affected, 161 (79.3%) agreed that regular eye checks are necessary, and more than half (54.2%) knew that diabetes can lead to blindness. Age, gender, level of education, and whether a participant or participant's friends and relatives had been diagnosed with diabetes were significantly associated with the knowledge and awareness of participants about diabetes and diabetic retinopathy.

**Conclusion:**

A majority of the participants had good knowledge of diabetes and diabetic retinopathy. Health education and promotion will also help increase the awareness of diabetes and diabetic retinopathy in the country.

## 1. Introduction

The prevalence of diabetes mellitus (DM) in the Pacific Island region is increasing, with urban migration as the leading cause [1]. In 2021, the International Diabetes Federation estimated that 51.5% of people with diabetes in Papua New Guinea (PNG) remain undiagnosed [2]. Individuals with diabetes tend to develop diabetic retinopathy (DR) over time due to poor regulation of blood sugar levels and the long duration of the condition [3]. DR is a common microvascular complication of DM, and it is a significant cause of visual impairment and blindness worldwide [4–7]. Therefore, regular screening of the eye is necessary to prevent visual impairment and blindness from DM. In order to make such screening programs effective and for people to actively take up services, the public needs to be aware and know about diabetes and its ocular complications.

Several research studies have been conducted in different parts of the world to assess the knowledge, awareness, attitude, perception, and practice towards DM and DR in a quest to strengthen eye care services [7–11]. To date, a few studies have assessed the prevalence of DM and its complications in PNG. Lesley et al. [12] indicated in their survey in Madang Provincial Hospital and fifteen other hospitals in the country that there was limited testing for complications of DM in PNG, and tests such as HbA1c were available in only one hospital. They reported that even though oral hypoglycaemic drugs and insulin were mostly available in hospitals, there was inadequate trained DM professionals such as endocrinologists and dieticians. Most recently, in 2017, a study was conducted by Burnett et al. [13] to assess the prevalence of DM and DR in PNG. The study estimated the prevalence of DM to be 8.1% out of which 62.5% were undiagnosed prior to the study. The study further posited that more than three-quarters of people with known diabetes never had an eye examination before the study, and nearly half of the participants with diabetes had developed diabetic retinopathy and/or maculopathy. Nonetheless, the study was conducted among the aged (50 years and above) and focused solely on the population in the National Capital District. Moreover, the study did not explore the participants' knowledge and awareness about the disease and its complications.

Currently, there are seventy-five (75) ophthalmic clinicians in PNG serving as the primary eye care workforce for the entire country. These clinicians are trained to identify and refer patients with DM and DR to the main centers for further assessment and intervention. The Madang Provincial Hospital Eye Clinic (MPHEC) and two other hospitals in the national capital are the only facilities which offer fundus photography and laser services for patients with DR in PNG as at the time of this study. In addition, the MPHEC provides routine blood glucose test for patients who are above 40 years of age. It is the only eye care facility in the province and serves as the center for training ophthalmic clinicians in the country. MPHEC is a free eye clinic, and all services are offered at no cost to the patients.

Despite the availability of free services, the uptake of DR screening at MPHEC is low at an average of two people per month since its inception in 2015. This and the aforementioned problems raised our interest to investigate the knowledge and awareness of DM and DR among ophthalmic patients in Madang Province in our quest to improve the delivery and uptake of health care services in the country. The outcome of this study will help policy makers and eye care service providers to streamline public education and design and implement appropriate services and professional training programs in the country.

## 2. Materials and Methods

### 2.1. Study Setting

This study was conducted at Madang Provincial Hospital Eye Clinic in Papua New Guinea. Madang Province is the fourth most populated province among the 22 provinces of Papua New Guinea [14]. The eye clinic is staffed with ophthalmic clinicians and an ophthalmologist, and they offer comprehensive services including fundus photography and laser treatment for DR. It is the only active eye clinic in the province and the services are provided at no cost to the patient; therefore, it is highly patronized by people in PNG.

### 2.2. Study Design and Sampling Techniques

This was a hospital-based cross-sectional study involving both qualitative and quantitative data. Convenience sampling was used to ascertain the level of awareness and knowledge of DM and DR by all patients who visited the eye clinic from July to August 2021.

### 2.3. Inclusion and Exclusion Criteria

The study included all patients who visited the eye clinic during the period of the study but excluded minors (≤17 years) and those who did not give consent to participate in the study.

### 2.4. Ethical Consideration

Ethical approval was obtained from the Faculty of Medicine and Health Sciences Research Committee (FMHSRC) of Divine Word University (reference number FRC/MHS/56-21), and the study adhered to the Declaration of Helsinki.

### 2.5. Data Collection Procedure

A structured questionnaire (see appendix (available (here))) was designed based on previous similar studies [7, 15], and it consisted of two parts: the first assessed basic socio-demographic and health information including gender, age, occupation, level of education, and history of DM. The second part contained nine questions and assessed the participants' knowledge of DM and DR. The preamble of the questionnaire contained information about the purpose of the study, an informed consent statement, and the participants' right to withdraw from the study. The questionnaire was developed in English and was administered by two of the investigators. The investigators interpreted the questionnaire in Tok Pidgin (the local language) for participants who could neither read nor understand English after which their responses were recorded by the investigators. No personally identifiable information was collected. A copy of the questionnaire is attached in Appendix 1.

### 2.6. Data Management and Analysis

All the returned questionnaires were secured with a lock, and only the investigators had access to them. Soft copies of data were stored on a password protected computer. GraphPad Prism version 9.3.1 (GraphPad Software, Inc., San Diego, California) was used for statistical analyses. Categorical and nominal data were summarized as percentages and frequencies, and the continuous variable, age, was presented as median (interquartile range). Normality was tested using Shapiro–Wilk's test, and comparisons were drawn using the Mann–Whitney *U*-test. The level of significance was set at *P* < 0.05.

## 3. Results

### 3.1. Characteristics of Participants

A total of 203 (97.6%) patients consented to and participated in the study out of 208 patients who were approached. The age of participants ranged from 19 to 78 years with a median (interquartile range) of 41 (53–29) years. 107 were females (52.7%), and 96 (47.3%) were males. A majority (31.5%) of the participants had tertiary education followed by secondary (31.0%). Among the participants, 8 (3.9%) were people with diabetes and 134 (66%) had no relatives or friends with DM. The most commonly reported occupations were farmers (22.2%), traders (14.8%), and students (12.8%).

### 3.2. Knowledge and Awareness of DM and DR

A total of 145 (71.4%) of the participants knew that diabetes can affect the eye. A majority (93.6%) of the participants checked their eyes only when their vision was affected, 161 (79.3%) agreed that regular eye checks are necessary and 110 (54.2%) knew that diabetes can lead to blindness as shown in Table 1. Significantly more females were aware that diabetic retinopathy can lead to blindness (*P* = 0.012). However, more males than females frequently checked their eyes (*P* = 0.035). From Table 2, the older patients usually depended on friends and relatives for information about DM and DR, while the younger patients mostly relied on the Internet for such information (*P* = 0.031). In addition, age was significantly associated with participant's reasoning on whether DR treatment can restore normal eyesight (*P* = 0.030).

Participants who had at least secondary education were more likely to indicate that patients with DM should seek regular eye check-ups at shorter intervals as reported in Table 3 (*P*=0.003). Again, participants with higher level of education mostly thought that blood glucose control can reduce the risk of DR (*P*=0.018). Significantly more participants in this group relied on different means especially the Internet to acquire information about DM and DR (*P* < 0.001). Furthermore, significantly more patients in this group indicated that lack of information was the main barrier to the uptake of regular eye check-ups (*P*=0.030).

In this study, participants with diabetes significantly indicated that they seek eye care services at shorter time intervals than the response obtained from participants without diabetes (*P*=0.005, Table 4).

The study further assessed whether there was any significant difference in the response between patients with or without close relations with diabetes as illustrated in Table 5. Participants who had relatives or friends with diabetes were more aware that diabetes can affect the eye (*P*=0.014). Significantly more of these same participants than participants without close relations with diabetes indicated that people with diabetes need regular eye examinations (*P*=0.010). In addition, they were more likely to recommend shorter time intervals of eye check-ups for people with diabetes (*P*=0.006). Moreover, patients who had close relations with diabetes mostly indicated that blood glucose control can reduce the risk of DR (*P*=0.036).

## 4. Discussion

DR is a complication of diabetes that can cause blindness and visual impairment [3]. A low proportion of the participants (3.9%) was known patients with DM, and 71.4% knew the effect of DM on the eyes. In a previous study in PNG, Burnett et al. [13] estimated the prevalence of DM as 8.1% which was higher compared to this study. The difference can be attributed to the age range involved in both studies. The current study was among all age groups, whilst the previous study was among participants aged 50 years and above. It is believed that aging is a risk factor for DM [16]; therefore, the incidence of DM is high among the aging population compared to those younger [17]. Furthermore, the previous study employed a population-based study design, and this current study was hospital-based.

A large number (54.2%) of the participants were aware that DR can lead to blindness, and this is higher than studies in Ethiopia [11] and India [10]. A study in Ethiopia among patients with diabetes reported a good knowledge of DR in 47.4% of the participants [11] and, similarly, in India, 47% of the participants had good knowledge of DR [10]. In contrast, studies in Saudi Arabia [9] and Bangladesh [18] reported higher values (64% and 76%, respectively). Organization of health talk shows and awareness campaigns may have contributed to the good knowledge level among the participants.

In this study, a higher number (79.3%) of the participants were aware of the need for people with diabetes to go for regular eye screenings. In comparison, other studies have reported relatively lower knowledge among participants on the regulation of blood sugar and its effect on the eyes: India [10] (33.7%), Malaysia [19] (50%), Kenya [20] (50%), Ghana [21] (65.4%), and Australia [22] (71%). Almost all patients (93.6%) in this study indicated that they seek eye care services only when their vision is affected, and a quarter (24.6%) suggested that people with diabetes should go for check-ups only when their vision is affected. However, this was lower than the responses in a hospital-based study among DM patients in Northwest Ethiopia [11] and a similar study in India [15]. In this study, people with diabetes significantly indicated that they seek eye care services at shorter time intervals than the response obtained from people without diabetes (Table 4). However, both groups of participants showed no significant difference in responding to their thought on how often people with diabetes should seek eye check-ups. There is therefore the need for proper education for people to understand the frequency and purpose of follow-up visits required for people with diabetes.

Nearly half of the participants (45.8%) did not know that DR can lead to blindness. This lack of knowledge may lead to poor attitude and practice towards seeking eye care services including DR care and management in PNG. Many studies have shown that some causes of visual impairment and blindness are asymptomatic until the diseases have progressed to an advanced state [6, 15, 23–25], and this often leads to irreversible blindness. Therefore, early diagnosis and treatment are essential to prevent sight loss from DR and other ocular diseases. The frequency of follow-up visits is an essential part in the management of DM and DR. It is recommended that people with diabetes attend DR screenings at the time of diagnosis and annually thereafter [26].

The study further probed to determine the reasons why people with diabetes in PNG do not attend regular eye check-ups. The lack of information on DM and DR at the community level (57.1%), fear of discovering something bad during medical checkups (16.3%), and distance (15.8%) were the main barriers to the uptake of regular eye examinations as reported by participants in this study. A majority of the participants relied on health facilities (42.9%) and close relations such as friends and relatives (20.7%) for health information. Older patients mostly relied on close relations while younger patients often accessed the Internet for health information (*P*=0.031). In addition, the more educated participants often sought health information from the Internet (*P* < 0.001). Patients who had relatives or friends with diabetes were more aware that diabetes can affect the eye, and they mostly indicated that people with diabetes need periodic eye examinations every half-year or annually. Furthermore, most of them knew that blood glucose control may reduce the risk of DR (Table 5). These findings suggest that the use of close relations and electronic media is reliable means to educate the public and increase the uptake of DM and DR services.

Nonetheless, more than one-fifth of the participants (20.7%) did not have access to health information. Health education and increasing accessibility to eye care services especially for the less privileged population will help eliminate these daring influences on seeking health information and checkups [11, 27]. These measures will be very essential since it is expected that noncommunicable diseases (NCDs) will be on the rise in PNG due to high levels of NCD risk factors [28] and the high level of undiagnosed diabetes in PNG [1] which could lead to visual impairment and loss of productivity.

This study was biased towards patients who visited the eye clinic for services. It is possible that the knowledge and awareness in the various communities of Madang Province may be worse than that of the patients. Therefore, the study findings cannot be fully generalized. Nonetheless, it was useful in providing the fundamental insights into the knowledge and awareness of DM and DR in the province. The study findings strongly suggest that removing barriers especially by improving health education in the communities can lead to increased uptake of DM and DR services. It further highlighted that practice-oriented education is an essential component of health promotion and treatment of diseases.

## 5. Conclusion

In conclusion, there was a good awareness of diabetes and diabetic retinopathy among the participants, but there remain many aspects which need improvements. Exposure to health care information from health facilities is one of the contributing factors towards the good knowledge among the participants in this current study. In addition, the target population might have influenced the good knowledge level among the participants in this study. It is recommended that policymakers and health authorities coordinate with media houses to increase awareness and knowledge of NCDs such as DM and DR. Proper knowledge and a positive attitude towards service delivery are essential pillars in eliminating blindness. A community-level study is encouraged to understand the knowledge and awareness of DM and DR among the general population and health workers in PNG.

## Figures and Tables

**Table 1:** Association between gender and knowledge and awareness of DM and DR.

Variables		Gender of patients (%)		Total (%)	*P* value
		Male	Female		
Can diabetes affect the eye or vision?	Yes	69 (34.0)	76 (37.4)	145 (71.4)	>0.999
	No	27 (13.3)	31 (15.3)	58 (28.6)	

How often do you check your eyes?	Only when vision is affected	86 (42.4)	104 (51.2)	190 (93.6)	0.035
	Yearly	6 (3.0)	1 (0.5)	7 (3.4)	
	Every 2 years	3 (1.5)	1 (0.5)	4 (2.0)	
	Every 6 months	1 (0.5)	1 (0.5)	2 (1.0)	

Do you think that regular eye check-ups are necessary for diabetic patients?	Yes	74 (36.5)	87 (42.9)	161 (79.3)	0.491
	No	22 (10.8)	20 (9.9)	42 (20.7)	

How often do you think a diabetic patient should go for eye check-ups?	Every 6 months	61 (30.0)	60 (29.6)	121 (59.6)	0.298
	Only when vision is affected	22 (10.8)	28 (13.8)	50 (24.6)	
	Yearly	13 (6.4)	15 (7.4)	28 (13.8)	
	Every 2 years	0 (0.0)	4 (2.0)	4 (2.0)	

Can an individual with controlled diabetes avoid regular eye check-ups?	Yes	35 (17.2)	29 (14.3)	64 (31.5)	0.234
	No	61 (30.0)	78 (38.4)	139 (68.5)	

Are you aware that diabetic retinopathy can lead to blindness?	Yes	43 (21.2)	67 (33.0)	110 (54.2)	0.012
	No	53 (26.1)	40 (19.7)	93 (45.8)	

Do you think that blood sugar control may reduce the risk of diabetic retinopathy?	Yes	75 (36.9)	80 (39.4)	155 (76.4)	0.622
	No	21 (10.3)	27 (13.3)	48 (23.6)	

Can diabetic retinopathy treatment restore normal eyesight?	Yes	63 (31.0)	62 (30.5)	125 (61.6)	0.312
	No	33 (16.3)	45 (22.2)	78 (38.4)	

Where do you mostly obtain your information about diabetes and diabetic retinopathy?	Hospital or eye clinic	39 (19.2)	48 (23.6)	87 (42.9)	0.308
	Friends and relatives	25 (12.0)	17 (8.4)	42 (20.7)	
	No information	18 (8.9)	24 (11.8)	42 (20.7)	
	The Internet	11 (5.4)	17 (8.4)	28 (13.8)	
	Other sources	3 (1.5)	1 (0.5)	4 (2.0)	

Why do you think people with diabetes do not attend regular eye check-ups?	Lack of information	58 (28.6)	58 (28.6)	116 (57.1)	0.529
	Fear of discovering something bad	8 (3.9)	25 (12.3)	33 (16.3)	
	Cost of test	6 (3.0)	4 (2.0)	10 (4.9)	
	Living in remote areas	18 (8.9)	14 (6.9)	32 (15.8)	
	Lack of time	5 (2.5)	5 (2.5)	10 (4.9)	
	Other reasons	1 (0.5)	1 (0.5)	2 (1.0)	

Total		**96 (47.3)**	**107 (52.7)**	**203 (100.0)**	

**Table 2:** Association between age and knowledge and awareness of DM and DR.

Variables		Age of patients in years (%)		Total (%)	*P* value
		≤40	≥41		
Can diabetes affect the eye or vision?	Yes	76 (37.4)	69 (34.0)	145 (71.4)	0.121
	No	23 (11.3)	35 (17.2)	58 (28.6)	

How often do you check your eyes?	Only when vision is affected	92 (45.3)	98 (48.3)	190 (93.6)	0.692
	Yearly	4 (2.0)	3 (1.5)	7 (3.4)	
	Every 2 years	2 (1.0)	2 (1.0)	4 (2.0)	
	Every 6 months	1 (0.5)	1 (0.5)	2 (1.0)	

Do you think that regular eye check-ups are necessary for diabetic patients?	Yes	80 (39.4)	81 (39.9)	161 (79.3)	0.729
	No	19 (9.4)	23 (11.3)	42 (20.7)	

How often do you think a diabetic patient should go for eye check-ups?	Every 6 months	55 (27.1)	66 (32.5)	121 (59.6)	0.155
	Only when vision is affected	30 (14.8)	20 (9.9)	50 (24.6)	
	Yearly	13 (6.4)	15 (7.4)	28 (13.8)	
	Every 2 years	1 (0.5)	3 (1.5)	4 (2.0)	

Can an individual with controlled diabetes avoid regular eye check-ups?	Yes	35 (17.2)	29 (14.3)	64 (31.5)	0.236
	No	63 (31.0)	74 (36.5)	139 (68.5)	

Are you aware that diabetic retinopathy can lead to blindness?	Yes	57 (28.1)	53 (26.1)	110 (54.2)	0.398
	No	42 (20.7)	51 (25.1)	93 (45.8)	

Do you think that blood sugar control may reduce the risk of diabetic retinopathy?	Yes	72 (35.5)	83 (40.9)	155 (76.4)	0.252
	No	27 (13.3)	21 (10.3)	48 (23.6)	

Can diabetic retinopathy treatment restore normal eyesight?	Yes	53 (26.1)	72 (35.5)	125 (61.6)	0.030
	No	46 (22.7)	32 (15.8)	78 (38.4)	

Where do you mostly obtain your information about diabetes and diabetic retinopathy?	Hospital or eye clinic	44 (21.7)	43 (21.2)	87 (42.9)	0.031
	Friends and relatives	15 (7.4)	27 (13.3)	42 (20.7)	
	No information	21 (10.3)	21 (10.3)	42 (20.7)	
	The Internet	19 (9.4)	9 (4.4)	28 (13.8)	
	Other sources	0 (0.0)	4 (2.0)	4 (2.0)	

Why do you think people with diabetes do not attend regular eye check-ups?	Lack of information	50 (24.6)	66 (32.5)	116 (57.1)	0.090
	Fear of discovering something bad	17 (8.4)	16 (7.9)	33 (16.3)	
	Cost of test	4 (2.0)	6 (3.0)	10 (4.9)	
	Living in remote areas	20 (9.9)	12 (5.9)	32 (15.8)	
	Lack of time	8 (3.9)	2 (1.0)	10 (4.9)	
	Other reasons	0 (0.5)	2 (1.0)	2 (1.0)	

Total		**99 (48.8)**	**104 (51.2)**	**203 (100.0)**	

**Table 3:** Association between level of education of patients and their knowledge and awareness of DM and DR.

Variables		Level of education (%)		Total (%)	*P* value
		<Secondary	≥Secondary		
Can diabetes affect the eye or vision?	Yes	50 (24.6)	95 (46.8)	145 (71.4)	0.200
	No	26 (12.8)	32 (15.8)	58 (28.6)	

How often do you check your eyes?	Only when vision is affected	72 (35.5)	118 (58.1)	190 (93.6)	0.764
	Yearly	3 (1.5)	4 (2.0)	7 (3.4)	
	Every 2 years	0 (0.0)	4 (2.0)	4 (2.0)	
	Every 6 months	1 (0.5)	1 (0.5)	2 (1.0)	

Do you think that regular eye check-ups are necessary for diabetic patients?	Yes	55 (27.1)	106 (52.2)	161 (79.3)	0.073
	No	21 (10.3)	21 (10.3)	42 (20.7)	

How often do you think a diabetic patient should go for eye check-ups?	Every 6 months	36 (17.7)	85 (41.9)	121 (59.6)	0.003
	Only when vision is affected	27 (13.3)	23 (11.3)	50 (24.6)	
	Yearly	10 (4.9)	18 (8.9)	28 (13.8)	
	Every 2 years	3 (1.5)	1 (0.5)	4 (2.0)	

Can an individual with controlled diabetes avoid regular eye check-ups?	Yes	29 (14.3)	35 (17.2)	64 (31.5)	0.125
	No	46 (22.7)	91 (44.8)	139 (68.5)	

Are you aware that diabetic retinopathy can lead to blindness?	Yes	43 (21.2)	67 (33.0)	110 (54.2)	0.663
	No	33 (16.3)	60 (29.6)	93 (45.8)	

Do you think that blood sugar control may reduce the risk of diabetic retinopathy?	Yes	51 (25.1)	104 (51.2)	155 (76.4)	0.018
	No	25 (12.3)	23 (11.3)	48 (23.6)	

Can diabetic retinopathy treatment restore normal eyesight?	Yes	46 (22.7)	79 (38.9)	125 (61.6)	0.882
	No	30 (14.8)	48 (23.6)	78 (38.4)	

Where do you mostly obtain your information about diabetes and diabetic retinopathy?	Hospital or eye clinic	34 (16.7)	53 (26.1)	87 (42.9)	<0.001
	Friends and relatives	14 (6.9)	28 (13.8)	42 (20.7)	
	No information	24 (11.8)	18 (8.9)	42 (20.7)	
	The Internet	1 (0.5)	27 (13.3)	28 (13.8)	
	Other sources	3 (1.5)	1 (0.5)	4 (2.0)	

Why do you think people with diabetes do not attend regular eye check-ups?	Lack of information	35 (17.2)	81 (39.9)	116 (57.1)	0.030
	Fear of discovering something bad	15 (7.4)	18 (8.9)	33 (16.3)	
	Cost of test	6 (3.0)	4 (2.0)	10 (4.9)	
	Living in remote areas	14 (6.9)	18 (8.9)	32 (15.8)	
	Lack of time	5 (2.5)	5 (2.5)	10 (4.9)	
	Other reasons	1 (0.5)	1 (0.5)	2 (1.0)	

Total		**76 (37.4)**	**127 (62.6)**	**203 (100.0)**	

**Table 4:** Association between the diabetic status of patients and their knowledge and awareness of DM and DR.

Variables		Are you a diabetic patient?		Total (%)	*P* value
		Yes	No		
Can diabetes affect the eye or vision?	Yes	8 (3.9)	137 (67.5)	145 (71.4)	0.108
	No	0 (0.0)	58 (28.6)	58 (28.6)	

How often do you check your eyes?	Only when vision is affected	5 (2.5)	185 (91.1)	190 (93.6)	0.005
	Yearly	1 (0.5)	6 (3.0)	7 (3.4)	
	Every 2 years	1 (0.5)	3 (1.5)	4 (2.0)	
	Every 6 months	1 (0.5)	1 (0.5)	2 (1.0)	

Do you think that regular eye check-ups are necessary for diabetic patients?	Yes	8 (3.9)	153 (75.4)	161 (79.3)	0.210
	No	0 (0.0)	42 (20.7)	42 (20.7)	

How often do you think a diabetic patient should go for eye check-ups?	Every 6 months	5 (2.5)	116 (57.1)	121 (59.6)	0.690
	Only when vision is affected	1 (0.5)	49 (24.1)	50 (24.6)	
	Yearly	2 (1.0)	26 (12.8)	28 (13.8)	
	Every 2 years	0 (0.0)	4 (2.0)	4 (2.0)	

Can an individual with controlled diabetes avoid regular eye check-ups?	Yes	5 (2.5)	59 (29.1)	64 (31.5)	0.068
	No	3 (1.5)	136 (67.0)	139 (68.5)	

Are you aware that diabetic retinopathy can lead to blindness?	Yes	7 (3.4)	103 (50.7)	110 (54.2)	0.073
	No	1 (0.5)	92 (45.3)	93 (45.8)	

Do you think that blood sugar control may reduce the risk of diabetic retinopathy?	Yes	6 (3.0)	149 (73.4)	155 (76.4)	>0.999
	No	2 (1.0)	46 (22.7)	48 (23.6)	

Can diabetic retinopathy treatment restore normal eyesight?	Yes	6 (3.0)	119 (58.6)	125 (61.6)	0.491
	No	2 (1.0)	76 (37.4)	78 (38.4)	

Where do you mostly obtain your information about diabetes and diabetic retinopathy?	Hospital or eye clinic	4 (2.0)	83 (40.9)	87 (42.9)	0.160
	Friends and relatives	2 (1.0)	40 (19.7)	42 (20.7)	
	No information	0 (0.0)	42 (20.7)	42 (20.7)	
	The Internet	2 (1.0)	26 (12.8)	28 (13.8)	
	Other sources	0 (0.0)	4 (2.0)	4 (2.0)	

Why do you think people with diabetes do not attend regular eye check-ups?	Lack of information	3 (1.5)	113 (55.7)	116 (57.1)	0.233
	Fear of discovering something bad	0 (0.0)	33 (16.3)	33 (16.3)	
	Cost of test	2 (1.0)	8 (39.4)	10 (4.9)	
	Living in remote areas	3 (1.5)	29 (14.3)	32 (15.8)	
	Lack of time	0 (0.0)	10 (4.9)	10 (4.9)	
	Other reasons	0 (0.0)	2 (1.0)	2 (1.0)	

Total		**8 (3.9)**	**195 (96.1)**	**203 (100.0)**	

**Table 5:** Association between the diabetic status of patients' close relations and their knowledge and awareness of DM and DR.

Variables		Is any of your relatives or friends a diabetic patient?		Total (%)	*P* value
		Yes	No		
Can diabetes affect the eye or vision?	Yes	57 (28.1)	88 (43.3)	145 (71.4)	0.014
	No	12 (5.9)	46 (22.7)	58 (28.6)	

How often do you check your eyes?	Only when vision is affected	62 (30.5)	128 (63.1)	190 (93.6)	0.079
	Yearly	5 (24.6)	2 (1.0)	7 (3.4)	
	Every 2 years	1 (0.5)	3 (1.5)	4 (2.0)	
	Every 6 months	1 (0.5)	1 (0.5)	2 (1.0)	

Do you think that regular eye check-ups are necessary for diabetic patients?	Yes	62 (30.5)	99 (48.8)	161 (79.3)	0.010
	No	7 (3.4)	35 (17.2)	42 (20.7)	

How often do you think a diabetic patient should go for eye check-ups?	Every 6 months	48 (23.6)	73 (36.0)	121 (59.6)	0.006
	Only when vision is affected	7 (3.4)	43 (21.2)	50 (24.6)	
	Yearly	13 (6.4)	15 (7.4)	28 (13.8)	
	Every 2 years	1 (0.5)	3 (1.5)	4 (2.0)	

Can an individual with controlled diabetes avoid regular eye check-ups?	Yes	20 (9.9)	44 (21.7)	64 (31.5)	0.634
	No	49 (24.1)	88 (43.3)	139 (68.5)	

Are you aware that diabetic retinopathy can lead to blindness?	Yes	41 (20.2)	69 (34.0)	110 (54.2)	0.301
	No	28 (13.8)	65 (32.0)	93 (45.8)	

Do you think that blood sugar control may reduce the risk of diabetic retinopathy?	Yes	59 (29.21)	96 (47.3)	155 (76.4)	0.036
	No	10 (4.9)	38 (18.7)	48 (23.6)	

Can diabetic retinopathy treatment restore normal eyesight?	Yes	46 (22.7)	79 (38.9)	125 (61.6)	0.292
	No	23 (11.3)	55 (27.1)	78 (38.4)	

Where do you mostly obtain your information about diabetes and diabetic retinopathy?	Hospital or eye clinic	31 (15.3)	56 (27.6)	87 (42.9)	0.348
	Friends and relatives	17 (8.4)	25 (12.3)	42 (20.7)	
	No information	10 (4.9)	32 (15.8)	42 (20.7)	
	The Internet	10 (4.9)	18 (8.9)	28 (13.8)	
	Other sources	1 (0.5)	3 (1.5)	4 (2.0)	

Why do you think people with diabetes do not attend regular eye check-ups?	Lack of information	41 (20.2)	75 (36.9)	116 (57.1)	0.936
	Fear of discovering something bad	11 (5.4)	22 (10.8)	33 (16.3)	
	Cost of test	3 (1.5)	7 (3.4)	10 (4.9)	
	Living in remote areas	13 (6.4)	19 (9.5)	32 (15.8)	
	Lack of time	1 (0.5)	9 (4.4)	10 (4.9)	
	Other reasons	0 (0.0)	2 (1.0)	2 (1.0)	

Total		**69 (34.0)**	**134 (66.0)**	**203 (100.0)**	

## Data Availability

Data are available upon appropriate request from the authors and the Faculty of Medicine and Health Sciences Research Committee (FMHSRC) of Divine Word University, Madang. The chair of FMHSRC can be contacted at eschuele@dwu.ac.pg.

## Supplementary Materials

Supplementary Materials Awareness and knowledge of diabetic retinopathy (DR) among patients attending at Madang Provincial Hospital Eye Clinic.
