# Supplementary material for: Knowledge and Awareness of Diabetes and Diabetic Retinopathy among Patients Seeking Eye Care Services in Madang Province, Papua New Guinea
Source: J Ophthalmol. 2022 Jun 1;2022:7674928. doi: 10.1155/2022/7674928 (PMC9177315; doi:10.1155/2022/7674928)
Supplement: Supplementary Materials — Awareness and knowledge of diabetic retinopathy (DR) among patients attending at Madang Provincial Hospital Eye Clinic. [file 7674928.f1.docx]

**Supplementary Material**

**Questionnaire**

**Awareness and knowledge of diabetic retinopathy (DR) among patients attending at Madang Provincial Hospital Eye Clinic**

**PART ONE: BASIC SOCIO-DEMOGRAPHIC INFORMATION**

Gender: M / F Age:­­­______ Occupation:___________ Level of Education: ______________

1. Are you a diabetic patient? □ Yes □No
2. Is any of your a relative or friends a diabetic patient? □ Yes □ No
3. How often do you check your eyes?

□Every 6months □Yearly □ Every 2years □Only when the vision is affected.

**PART TWO: KNOWLEDGE ABOUT DIABETES AND DIABETIC RETINOPATHY**

1. Can diabetes affect the eye or vision? □ Yes □No
2. Do you think that regular eye check-ups are necessary for diabetic patients? □ Yes □No
3. How often do you think a diabetic patient should go for eye check-ups?

□Every 6months □Yearly □ Every 2years □Only when the vision is affected.

1. Can an individual with controlled diabetes avoid regular eye check-ups? □ Yes □No
2. Are you aware that diabetic retinopathy can lead to blindness? □ Yes □No
3. Do you think that blood sugar control may reduce the risk of diabetic retinopathy? □ Yes □No
4. Can diabetic retinopathy treatment restore normal eyesight? □ Yes □No
5. Where do you mostly obtain information about diabetes and diabetic retinopathy?

□The internet □Magazines □ Eye clinic/hospital □Friends and relatives

□I do not get any information □Other sources (please specify) ________________________

1. Why do you think people with diabetes do not attend regular eye check-ups?

□Lack of information □Lack of time □ Cost of test □Fear of discovering something bad

□Living in remote areas □Other reasons (please specify) ________________________

End of questionnaire……………….Thank you.
